# Supplementary material for: Co-Cultures of Lactobacillus acidophilus and Bacillus subtilis Enhance Mucosal Barrier by Modulating Gut Microbiota-Derived Short-Chain Fatty Acids
Source: Nutrients. 2022 Oct 25;14(21):4475. doi: 10.3390/nu14214475 (PMC9657225; doi:10.3390/nu14214475)
Supplement: Supplementary file 1 [file nutrients-14-04475-s001.zip › nutrients-1948411-supplementary.pdf]

Supplementary Table S1. Primer sequences used in the qRT-PCR experiment

Gene	Primer Sequences (5'-3')
AhR	F: ACTACCACCCATCTTCACCCG<br>R: CAACACACATCAATGCTTCCC
GAPDH	F: CCAGGGCTGCTTTTAACTCTG<br>R: GTGGGTGGAATCATACTGGAACAT
GPR41	F: TACTCGCGCTTTGTGTGGAT<br>R: CATTTGGGGCTCTCCTTCGT
GPR43	F: TGCAGCGTGGTCTACATCAT<br>R: AAAGGACCACAGCCATCTCC
GPR109A	F: GAGTTTGTGTTCGGGCTCCT<br>R: GAGCATTATTCGGCAGGGGA
IL-10	F: GCTGAAGACCCTCAGGCTGA<br>R: TTGCTCTTGTTTTCACAGGGC
IL-22	F: CCTACATCACCAACCGCACCT<br>R: CTGATTGACTCCCTGGAACAGATTA
NF-κB	F: CTCGCACAAGGAGACATGAA<br>R: ACTCAGCCGGAAGGCATTAT
PBD-2	F: ACCTGCTTACGGGTCTTG<br>R: CTCTGCTGTGGCTTCTGG
PBD-3	F: GAAGTCTACAGAAGCCAAAT<br>R: GGTAACAAATAGCACCATAA
RegIII γ	F: AACCTGGATGGGTGCAGACGTG<br>R: AACCTGGATGGGTGCAGACGTG
TNF-α	F: CCAATGGCAGAGTGGGTATG<br>R: TGAAGAGGACCTGGGAGTAG

A

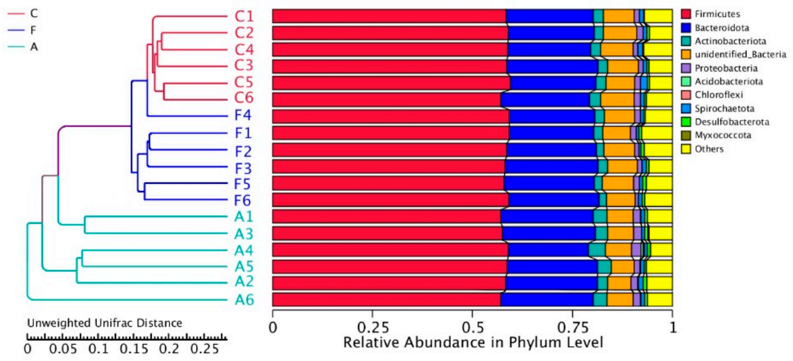

B

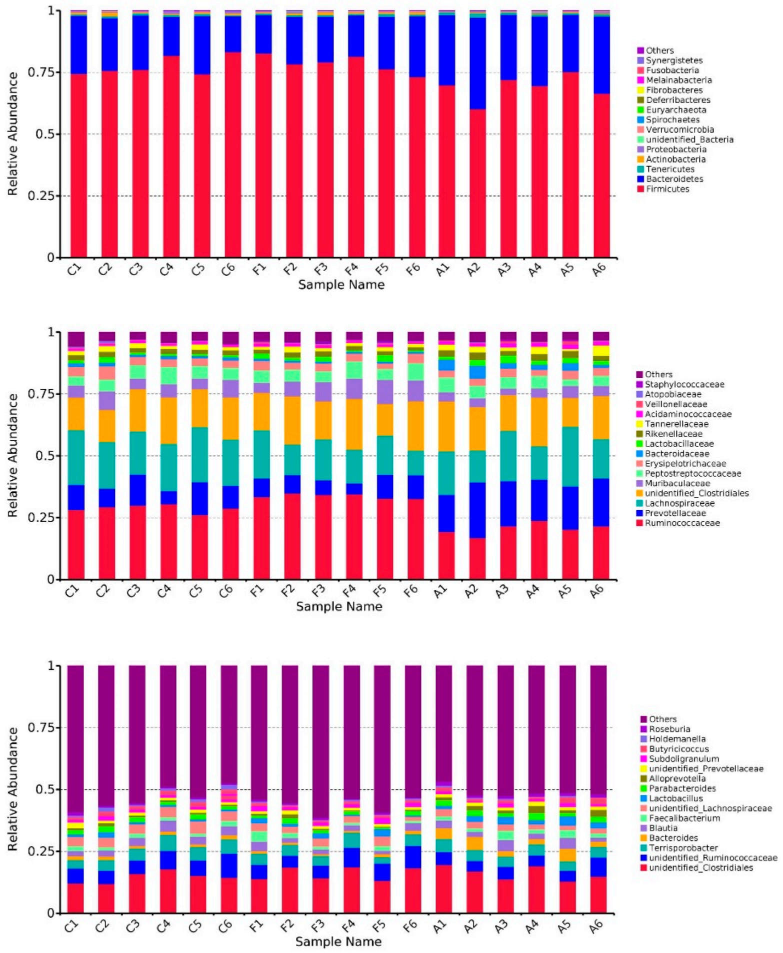

Supplementary Figure S1. Relative abundance of intestinal microbiota at the phylum level. (A) The cluster tree of microbial communities in phylum level were obtained by UPGMA Cluster Analysis based on Unweighted Unifrac distance. (B) Relative abundance of bacterial communities in colon

among individuals at the phylum level (top), family (middle) and genus (bottom) level. C: control group, F: FAM group, A: antibiotic group.
